# An Opinion Paper on Aerogels for Biomedical and Environmental Applications

**DOI:** 10.3390/molecules24091815

**Published:** 2019-05-10

**Authors:** Carlos A. García-González, Tatiana Budtova, Luisa Durães, Can Erkey, Pasquale Del Gaudio, Pavel Gurikov, Matthias Koebel, Falk Liebner, Monica Neagu, Irina Smirnova

**Affiliations:** 1 Department of Pharmacology, Pharmacy and Pharmaceutical Technology, R+D Pharma group (GI-1645), Faculty of Pharmacy and Health Research Institute of Santiago de Compostela (IDIS), Universidade de Santiago de Compostela, E-15782 Santiago de Compostela, Spain; 2 MINES ParisTech, PSL Research University, CEMEF – Center for materials forming, UMR CNRS 7635, CS 10207, 06904 Sophia Antipolis, France; tatiana.budtova@mines-paristech.fr; 3 CIEPQPF, Department of Chemical Engineering, University of Coimbra, Rua Sílvio Lima, 3030-790 Coimbra, Portugal; luisa@eq.uc.pt; 4 Department of Chemical and Biological Engineering, Koç University, Sariyer, Istanbul 34450, Turkey; cerkey@ku.edu.tr; 5 Department of Pharmacy, University of Salerno, I-84084 Fisciano (SA), Italy; pdelgaudio@unisa.it; 6 Institute of Thermal Separation Processes, Hamburg University of Technology, Eißendorfer Straße 38, 21073 Hamburg, Germany; pavel.gurikov@tuhh.de (P.G.); irina.smirnova@tuhh.de (I.S.); 7 Laboratory for Building Energy Materials and Components, Swiss Federal Laboratories for Materials Science and Technology - Empa, Überlandstrasse 129, CH-8600 Dübendorf, Switzerland; matthias.koebel@empa.ch; 8 Institute for Chemistry of Renewable Resources, University of Natural Resources and Life Sciences Vienna, Konrad-Lorenz-Straße 24, 3430 Tulln an der Donau, Austria; falk.liebner@boku.ac.at; 9 Immunology Department, “Victor Babes” National Institute of Pathology, 99-101 Splaiul Independentei, 050096 Bucharest, Romania; neagu.monica@gmail.com

**Keywords:** aerogels, biomedical applications, environmental applications, bio-based aerogels, circular economy, biorefinery, active ageing

## Abstract

Aerogels are a special class of nanostructured materials with very high porosity and tunable physicochemical properties. Although a few types of aerogels have already reached the market in construction materials, textiles and aerospace engineering, the full potential of aerogels is still to be assessed for other technology sectors. Based on current efforts to address the material supply chain by a circular economy approach and longevity as well as quality of life with biotechnological methods, environmental and life science applications are two emerging market opportunities where the use of aerogels needs to be further explored and evaluated in a multidisciplinary approach. In this opinion paper, the relevance of the topic is put into context and the corresponding current research efforts on aerogel technology are outlined. Furthermore, key challenges to be solved in order to create materials by design, reproducible process technology and society-centered solutions specifically for the two abovementioned technology sectors are analyzed. Overall, advances in aerogel technology can yield innovative and integrated solutions for environmental and life sciences which in turn can help improve both the welfare of population and to move towards cleaner and smarter supply chain solutions.

## 1. Introduction: The Relevance of Nanostructured Materials in the Current Biomedical and Environmental Scenario

Current challenges in biomedicine are interlinked to the new socio-sanitary needs worldwide derived from the dramatic demographic changes and a new population lifestyle taking shape [1]. Namely, the population in Europe is experiencing a marked increase in longevity with 18.5% of people above 65 years of age already in 2014 and a prospect of 29.5% by 2060 [2]. New materials and approaches should provide solutions to enhance the quality of life and well-being at least at the same pace as the aging of the population and the prevalence of chronic diseases (cancer, cardiovascular, diabetes) rises. High-performance, reliable, safe and reproducible products are actively sought in response to the current demographic scenario of longevity by extending their lifetime efficiency and a more effective and responsible use of drugs (e.g., antibiotics). However, fragmented knowledge of the relevant interdisciplinary scientific domains (biomedicine, materials science, process engineering, regulatory aspects) may severely jeopardize the development of next-generation drugs and tissue grafts able to meet new social demands.

For environmental applications, the mandatory change towards a circular economy, implying a more sustainable management of resources, is one of the main pillars to be addressed in the current world context of ever-increasing energy (50% more by 2030 in Europe) and water (40%) demands [3,4,5,6]. Accordingly, European Union climate action targets for 2030 are a reduction of more than 40% in greenhouse gas emissions (in comparison to 1990), a minimum contribution of renewable energy of 32% to the total energy consumption and an increase in energy efficiency of at least 32.5% [7]. This new environment protecting policy is crucial for tackling the major ecological problems like global warming and desertification by soil erosion (deforestation) and avoiding potential risks of water and food shortages. In this context, international environment protecting strategies also pursue much better valorization of by-products and waste materials using improved biorefinery approaches. This new paradigm goes beyond environmental concerns and is part of a structural change in the global economy. Added-value innovative products and energy-efficient solutions are developed to mitigate external dependence and to maximize competitiveness, which in principle allows us to prolong economic growth and increase prosperity. The collaboration between all the actors along the value chain as well as a fruitful interaction with regulatory experts on circular economy is needed to promote this new environmentally conscious approach.

Nanostructured materials can actively contribute to solve the current demands in biomedicine and environmental remediation. Novel formulations and innovative materials with predictable morphologies and controlled properties are under development. In particular, nanostructured formulations retaining components previously approved by regulatory agencies are of particular interest for biomedical applications from regulatory and economical perspectives. Advanced nanostructured carriers are under research in pharmaceutical technology to tackle current limitations in the formulation of proteins, cytotoxic drugs and poorly water-soluble drugs regarding stability enhancement, minimization of side effects and increased therapeutic effect. In regenerative medicine, novel synthetic nanostructured scaffolds have the prospect to overcome challenges linked to availability (more than 2·10^6^ procedures per year worldwide for bone grafting), slow recovery, infections and unwanted side effects up to rejection occurring with biological grafts, which are the current gold standard [8]. For wound healing, the use of mesoporous materials in medical devices can keep suitable exudate equilibrium at the wound site [9,10,11].

Innovations in nanostructured materials for low carbon emissions processes as well as for air and water treatments are sought to prevent health and environmental risks arising from pollutants (greenhouse gases, nanoparticles carried by flue gas, carcinogenic compounds, endocrine disruptors, heavy metals, sabotage agents or medicines with additive effects) in humans, biota and environment. These new solutions for efficient capture, neutralization or detoxification of pollutants should embrace all steps of the circular economy model: process innovations to mitigate emissions, novel analytical tools allowing faster detection and at lower concentrations, and the development of prompt and highly efficient remediation and recovery techniques.

Overall, current research efforts on nanostructured materials for biomedical and environmental applications have severe limitations in terms of yield, reproducibility and toxicity [12,13,14]. These pitfalls translate into cumbersome processing techniques and limited cost-efficiency that restricts the access to the market. Novel advanced nanostructured materials, particularly aerogel-based materials, are promising candidates to master these challenges and to circumvent the said limitations [15,16]. The relevance of aerogels can be ascertained by recent review articles [15,16,17,18,19,20,21,22,23,24,25] and two editorial projects [26,27], which summarize already extensive experimental data from research results and their analysis. Based on the most recent advances and uses in aerogel technologies, the anticipated future trends in aerogels applied to biomedical and environmental applications are presented in this opinion paper.

## 2. Current Status on Aerogels for Biomedical and Environmental Applications

Aerogels can be defined as solid, lightweight and coherent open porous networks of loosely packed, bonded particles or nanoscale fibers, obtained from a gel following the removal of the pore fluid without significant structural modification. Given the small feature sizes, aerogels are generally endowed with a very high specific surface area [28,29]. In addition, this special class also unites intriguing properties like high porosity, low bulk density, outstanding textural properties as well as tuneable surface chemistry in most cases [26,30]. Namely, the combination of low density and high mesoporosity (pore size 2–50 nm) of classical aerogels (e.g., silica) has been notably exploited for thermal insulation in building materials and aerospace technologies. Several products are already commercialized for these specific applications (for example insulating pipes/boards/blankets/ translucent panels).

**Figure 1:**
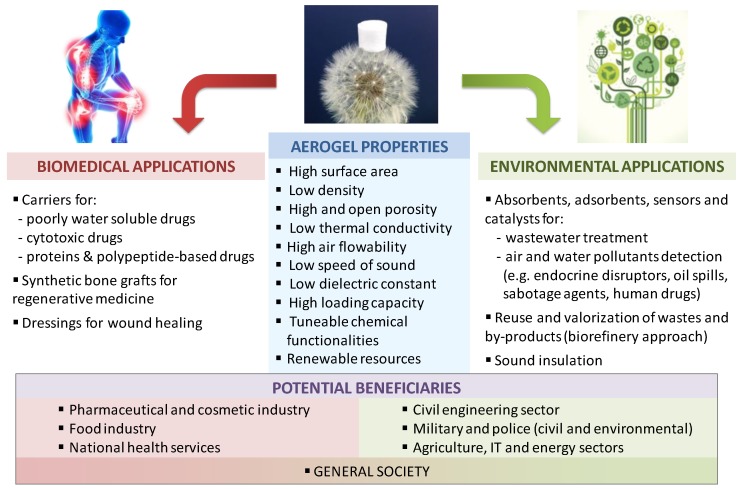
Outlook of advanced biomedical and environment applications of aerogels susceptible of being prospected in the future. Adapted from [31].

In biomedicine and environmental applications, the use of aerogel technology holds great promise to provide a compliant materials design platform in terms of yield, reproducibility and toxicity for satisfying the current social needs. The choice of the proper material science and technological approach to obtain an advanced material tailored for the envisaged biomedical or environmental application is claimed to give rise to important innovation breakthroughs in the field [31]. These advanced materials can be used in biomedical and environmental applications for several potential sectors (Figure 1).

**Figure 2:**
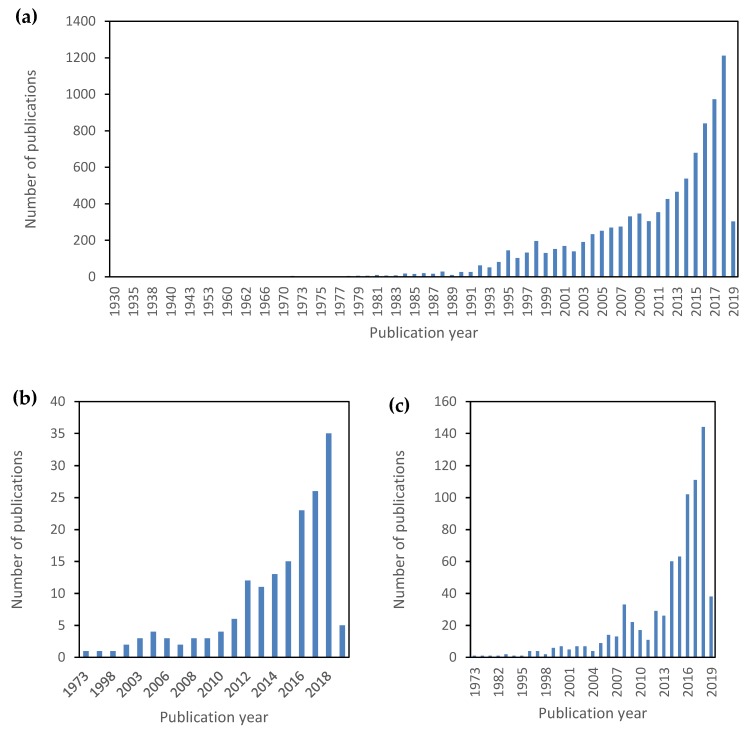
Trends in articles published worldwide on aerogels: (**a**) aerogels (search criteria: “aerogels”), (**b**) natural polymer-based aerogels (search criteria: “polysaccharide aerogels” OR “protein aerogels”), and (**c**) hybrid aerogels (search criteria: “hybrid aerogels”). Source: Scopus (search date: 26/02/2019).

Since their first invention in the 1930s, the interest in aerogels has significantly grown up, especially over the last two decades (Figure 2a). Traditional aerogels were almost exclusively obtained from alkali silicates, metal alkoxides (e.g., silica, boron) or low-molecular weight organic precursors capable of forming 3D networks upon polymerization (e.g., resorcinol-formaldehyde resins, polyurethane). Nowadays, biopolymers from polysaccharides or proteins are increasingly considered as promising sources of novel hybrid and composite aerogels [22,32,33,34,35]. This development is unambiguously reflected by the exponential increase in the respective publications in recent years (Figure 2b,c).

The survey of literature, patents and previous research on aerogels unveils biomedical and environmental applications as two important and prospective directions where aerogels have excellent upside potential. The main drawback to be tackled in aerogel technology to become a mainstream solution for biomedical and environmental applications is related to their price as the production of aerogels involves drying with supercritical CO_2_ and thus high-pressure technologies. In the case of synthetic polymer-based aerogels, the insufficient biodegradability of the material itself and toxic precursors and/or degradation products is also a problem to be solved. Finally, poor mechanical properties of certain inorganic aerogels are to be improved. Current approaches to circumvent or to solve these problems as well as current research uses of aerogels in biomedicine and for environmental applications are disclosed in the following paragraphs whereas the pending technological challenges are discussed in Section 3.

From a technological point of view, supercritical fluid-based drying of gels is regarded as the most suitable aerogel end-processing approach [19]. This gentle drying technique allows for far-reaching preservation of the initially formed fragile solid network structure. Other drying techniques (ambient drying, freeze-drying) can be, in certain cases, trade-off options to obtain xerogels and cryogels with comparable properties to aerogels depending on the gel source, the solid content and the mechanical stability of the 3D-structure as well as the target morphological, physicochemical and mechanical properties [36]. In the case of evaporative drying, additional steps are usually needed (solvent exchanges, silylation) to avoid the shrinkage of the fine porous structure. Optimization and integration of the supercritical drying process from an economic and environmental perspective as well as alternative drying processes are a current topic of great collaborative research and engineering efforts [37,38,39].

Biopolymers as renewable resources are of particular interest for biomedical and environmental applications due to their compatibility with living tissue (biocompatibility) and bio-degradability [18,21,40]. In this context, it has been demonstrated that abundant natural polymers like cellulose, starch or pectin can be transformed into high value-added gels and aerogels that have been promisingly tested as cell scaffolding materials, artificial cartilage, blood vessels or for efficient adsorption of noble metals (recycling) or pollutants of aqueous systems (e.g., heavy metals, oil, organic compounds) [25,41,42,43]. A significant advantage of bio-based aerogels over their synthetic polymer-based counterparts is that no toxic compound is involved in their preparation. Bio-based aerogels are thus “human-friendly” and can be directly used in life science applications. Novel bio-based aerogels targeting direction-dependent properties or using the particular self-assembling capabilities of nanoparticulate matter (e.g., nanocellulose) are expected to further broaden the application potential in biomedicine and separation science [17].

On the other hand, due to the chemical versatility of silicon chemistry, pure silica aerogels or silica-based aerogels are very common, but they show poor mechanical properties that compromise the durability of the material and restricts their applicability for uses requiring strength and stiffness. However, silica aerogels with improved mechanical properties (strength and/or flexibility) can be processed by the proper choice of silane precursors or by preparing hybrid (e.g., poly-organosiloxanes) and composite aerogels containing silica and organic polymers or fiber mats [44]. The use of natural fibers can be of particular interest to reinforce ambient pressure dried silica aerogel composites [45,46]. Another recent trend is the mechanical reinforcement of silica aerogels with carbon-based materials (carbon nanotubes, carbon aerogels of graphene) [47,48]. These silica aerogels and alternative aerogel sources (e.g., cellulose, polyurethane) may exhibit remarkable mechanical properties and endowed with supplementary properties like hierarchical porosity, shape memory and superflexibility [49,50,51].

Aerogels can find relevant and straightforward applications in biomedicine. For pharmaceutical applications, aerogels as drug delivery matrices for several administration routes (e.g., oral, pulmonary, nasal, topical) are able to improve the therapeutic outcome with modified drug release profiles. Therapeutic platforms based on aerogels are able to significantly improve drug bioavailability and to control the delivery of bioactive molecules [52]. Aerogels can be shaped “on demand” with various methods resulting in particle size varying from few microns to few millimeters [53]. For example, due to their high porosity, aerogel particles can act as dry powder carriers for pulmonary delivery in the form of particles with geometric diameters (20–25 µm) larger than 5 µm but still fitting to the required aerodynamic particle size distribution to penetrate in the lung’s alveoli [54]. The advantages of using biopolymer-based aerogels as carriers are as follows: improved dissolution rate of poorly water soluble drugs, high specific drug loadings, enhanced stability of amorphous drugs and excellent air flowability, which are requested for certain administration routes (oral, topical, pulmonary, nasal) [18,20,21,54,55,56,57,58,59,60,61,62]. Therefore, novel aerogel formulations would increase the therapeutic efficiency of the treatment (for example, improved lung deposition by oral inhalation, improved dissolution rate of poorly water-soluble drugs in oral drug delivery, enhanced drug permeation in nasal delivery) leading to economic savings and higher social adherence [1,14].

For wound healing, the use of aerogels allows the local formation of a wet gel into the lesion keeping proper exudate equilibrium at the wound, thus avoiding the traumatic removal from perilesional skin of conventional products. Biocompatible cellulose aerogels equipped with a short fluorescent peptide motive have been demonstrated to be promising protease sensing and sequestering dressings for chronic wounds [63,64]. The incorporation of aerogels in advanced dressings for wound healing [9,10,11] aims to become a high growth technology for the market of advanced bioactive dressings representing ca. 500 M€/yr worldwide and a top growth segment (expected annual growth: 15–25%) [65]. Such aerogel-based dressings can have a high impact in preventing or healing chronic wounds such as diabetic foot ulcers (15% incidence among diabetic patients and main cause of non-traumatic amputation of the lower extremity) or pressure ulcers (representing ca. 4% of total national sanitary costs) [66].

For regenerative medicine and plastic surgery, aerogel-based scaffolds have a nanostructure that can mimic the extracellular matrix of the natural tissue and aerogel-containing scaffolds lead to materials with improved roughness and pore interconnectivity of interest for tissue integration [8,16,41,51,67,68,69,70,71]. For example, cellulose phosphate of low degree of substitution has been demonstrated to be a good source for such cell scaffolding materials since it allows robust growth of mesenchymal stem cells, osteogenic differentiation, formation of hydroxyapatite layer in simulated body fluid, is hemocompatible and does not show an inflammatory response on the alternative pathway [72]. Cost-effective and safe aerogel scaffolds aim to give response to the availability problems and deficient tissue recovery of current grafts with a global market only for bone implants of ca. 5000 M€/yr [2,73]. Overall, advances from the use of aerogels may result in better clinical outcomes leading to reduced recovery time periods and the subsequent savings in direct (hospitalization) and indirect (sick leave and in-home healthcare expenses) cost of such treatment in the context of an aging global population—one of the primary drivers of burgeoning healthcare expenses.

For environmental applications, aerogels have already been produced from renewable resources (polysaccharides and proteins) thus participating to the sustainable bio-economy approach by obtaining specialty products from commodity natural polymers. Aerogels obtained using the biorefinery approach have been explored by means of the reuse or valorization of wastes (rice husk, wasted paper, waste biomass among other residues) and by-products (lignin) as gel sources or admixtures [67,74,75,76]. Physicochemical modifications of aerogels in terms of morphology and chemical functionalities are being actively prospected for environmental applications [22,23,77]. Thermal superinsulation is a particularly vivid field in bio-based aerogel research since respective aerogels with thermoconductivity values below that of air (26 mW m^−1^ K^−1^) have been obtained from different source materials, such as pectin [34,78] or cellulose [79]. There is a growing demand for new, cheaper and/or more efficient materials for water treatment and air/gas stream cleaning. Aerogel-based products are developed in the form of absorbents, adsorbents or catalysts and give improved response to the capture and/or degradation of oil spills and various toxic compounds (VOCs, heavy metals) [80,81,82,83,84,85,86,87,88]. The high specific surface area of mesoporous aerogels is of special interest for use in catalysis since values are comparable to common catalysts like microporous zeolites. Aerogel-based catalysts may prove superior in applications which target reducing greenhouse gas emissions and storage of solar energy such as hydrogen fuel cells and electrolyzers [89,90]. These aerogel-based materials for environmental applications should contribute to the reduction of water and air pollution, energy savings, responsible use of resources, fast detection of pollutants and the recovery of the compounds (aerogels; pollutants with economic value). They should also contribute to the reinvention of the global economy towards process innovations following the circular model of efficient and responsible management of resources and low carbon emissions estimated at 2.7 trillion € [91,92].

## 3. Prospects and Challenges in Aerogel Research

The current advance in aerogel research aspires to have a high scientific, technological and socioeconomic impact by generating added value solutions in terms of scientific knowledge, high-performance materials, and efficient, health-compliant and environmentally responsible technologies. The said impact is linked to the unresolved applications (chronic wound healing, cancer, bone scaffolds, sound insulation, air and water pollution monitoring and remediation) [93] to be targeted by aerogel technology and the subsequent socioeconomic relevance. The risk of feasibility can be considered as relatively low, since many of the desired properties for the end materials have been already individually achieved at laboratory scale. The risk of products and technologies being not cost-competitive for biomedical and environment applications (gross margins >60%) is also low since there are aerogel products in markets with lower venture profitability (e.g., building insulation, 20–30%) [94,95]. Moreover, like-for-like substitution of most products with aerogels using the same raw materials (polysaccharides, proteins) is possible without significant increase in operating costs, and the common solvents (water, alcohol, CO_2_) used for aerogel processing are already individually accepted by agencies thus reducing the associated risks and costs for regulatory compliance. The key missing factor is in many cases the awareness of aerogels in new market sectors. Three main issues on aerogel technology are generally recognized as main challenges of aerogel research, not fully explored yet and to be disclosed in the following subsections.

**Figure 3:**
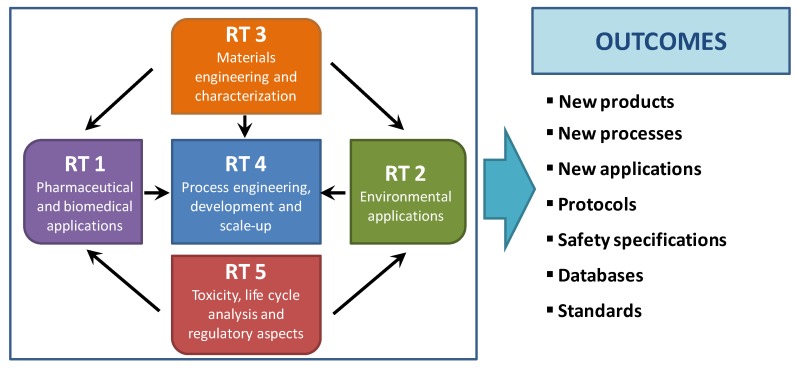
PERT chart for aerogels development in biomedical and environmental applications: research topics (RT, in text boxes), RT-interrelations (in arrows) and expected outcomes (right). Adapted from [31].

First, interdisciplinary approach is strongly needed to develop aerogels for targeted applications and to translate the aerogel advances and challenges into scientific, technological and regulatory outcomes as well as commercial products (Figure 3). Clusters should be established to identify the opportunities of aerogel technology to give response to the current social demands in the fields of biomedicine and environmental applications. Specific cutting-edge bioactive aerogels should be defined for biomedical applications as well as innovative aerogel features and products are needed for environmental applications, considering their respective market impacts. Then, process and materials engineering is needed to explore novel or modify existing chemical (sol-gel) and physical (e.g., drying) routes for aerogel processing, their scaling–up, to turn advanced materials development from lab-scale into commercial products considering technological, safety and economical aspects. Toxicity, health, environmental impact, life cycle analysis and regulatory aspects must be taken into account to successfully advance in these directions. Overall, a robust interdisciplinary scientific cooperation with the common scope of developing materials, technologies and protocols able to advance on the state-of-the-art of aerogels for environment and life sciences applications is necessary.

### 3.1. To Extend the Use of Aerogels for Biomedical and Environment Applications

For biomedical applications, the development of new aerogel formulations and the processing of innovative aerogel-containing dosage forms with selective response to external stimuli and with smart behaviour still have a large room for advancements. Aerogel formulations showing tunable drug release behaviour relevant to the intended administration route/target site, being able to deliver cytotoxic drugs (e.g., anti-cancer drugs) with enhanced therapeutic effect, or being compatible with the incorporation of proteins and polypeptide-based drugs are among the expected developments. Aerogel formulations with capacity of multiple release of different biomolecules is also currently an intense topic of research. From the point of view of the processing of aerogel-based drug products, the handling (powder flow properties, stability under storage), dosing (tableting), manufacturing (integrity under tableting, shaping of the aerogel morphology, choice of packaging) and validation of the aerogel dosage forms for different therapeutic applications and administration routes, as well as the implementation of the aerogel processing under Good Manufacturing Practices (GMP) conditions are to be endeavored.

For regenerative medicine, progresses on aerogels should be focused on the materials design to maximize osteointegration and on regulatory compliance. Accordingly, efforts should be devoted to get a robust processing method to confer a controlled macroporosity to the aerogels allowing host cell colonization, or promoting allo- and xenocells proliferation. Moreover, new aerogel synthesis methods should be engineered to improve the mechanical properties of the scaffolds and temporarily surrogate natural tissue without compromising the biocompatibility of the material. These processing methods should also allow the incorporation of bioactive compounds (e.g., growth factors) in high yields and with retained activity to promote the biological tissue growth [96]. From a regulatory point of view, the cytocompatibility and sterility of the aerogel-based materials should be predicted beforehand by developing precise methodologies able to ensure the presence of organic solvents or crosslinkers at concentrations below cytotoxic levels and to guarantee Sterility Assurance Level (SAL)-6 conditions for aerogel implants, as needed by regulatory agencies.

For the case of wound healing [66], the potential of aerogels as therapeutic platforms to promote and accelerate the natural wound healing process should be the subject of further research. Aerogels are to be explored to control a balanced exudate in the wound surroundings, to allow suitable gas permeability (transpiration) and to sustain the release of encapsulated bioactive compounds for certain applications (e.g., treatment of chronic wounds) and compared to benchmark products. In both wound healing and regenerative medicine, 3D-printing technique should be developed and adapted to “print” aerogels of complex shapes.

For environmental applications, the advances in aerogels should focus on materials (adsorbents, absorbents, sensors, catalysts) with a better performance than the current solutions (if any) in the market in terms of reliability, fast response, easy to use and recyclable [97]. Moreover, the following issues should also be taken into account (i) the search of new raw materials and feedstocks, (ii) a processing with a responsible management of resources, and/or (iii) a competitive production cost [98]. Accordingly, a critical study of aerogel sources (wastes and by-products) coming from the biorefinery approach should be further explored. Namely, novel routes for bio-based and hybrid aerogels, a third generation of aerogels based on polysaccharides and proteins, should be evaluated for various applications (acoustic and thermal insulation, absorption, adsorption, catalysis, active packaging). The screening, selection and testing of aerogel-based materials should be carried out targeting environmental applications like capture of spills, remediation of chemicals (air cleaning, water pollutant treatment and detection of contaminants) or thermal and sound insulation (to decrease thermal conductivity and to mitigate acoustic contamination in e.g., construction and transport applications) [22,23]. Novel surface modification techniques and alternative templating processing methods to synthesize aerogels with tunable surface chemistry and pore size should also be addressed to impart aerogels specific sorption (oil, heavy metals, organic pollutants) and/or capabilities for catalysis/electrochemical applications. The specific aerogel designs for the recovery of pollutants with economic interest as well as strategies for aerogel reuse should be especially considered among the forthcoming approaches. Moreover, new sensor solutions for the fast detection of air and water pollutants (endocrine disruptors, sabotage agents, metabolites, human and veterinary drugs, pesticides) with reduced sampling times and high sensitivities are also urgent environmental concerns that can be potentially tackled with aerogel-based solutions [18].

### 3.2. To Establish Novel Processing Routes and Designs, Robust Prediction Models and Characterization Tools for the Quality Control of Aerogels

Fundamental and applied research on aerogels for biomedical and environmental applications should be conducted to impart aerogels application-specific properties such as hydrophilicity, lipophilicity, ultra-low density, flame retardation, tailored porosity, nanomorphology and surface chemistry [99,100]. Fundamental research includes the study of biopolymer self-assembling in solution state, coagulation after anti-solvent addition and gel formation. Applied research comprises the development of aerogels in various shapes (monolithic blocks, particles, beads, fibers, films and scaffolds) with advanced properties for biomedical (e.g., drug delivery systems, medical devices in wound healing, scaffolds for regenerative medicine) and environmental (e.g., acoustic insulation, catalyst) applications [16,18,52,53]. Modelling and monitoring processing routes for their optimisation should also be considered.

Technological progresses should be aligned with these research advances. Design procedures for novel unit operations are to be developed with the goal to allow the manufacturing of different types of aerogels (inorganic, organic and hybrid) and preferably even of varying morphologies at pilot and later on industrial scale. The integration of multiple unit operations into one step and their optimization are key elements for minimizing CAPEX and OPEX costs of aerogel production. In certain cases, aerogel technology should be combined with other processing technologies (e.g., emulsion, plasma treatment, spray freeze-drying, supercritical foaming, 2D- and 3D-printing, jet cutting) or post-processing (jet milling, coating, post-impregnation) technologies [53,69,101,102,103] to get synergies and enhanced properties regarding mechanical, physicochemical, microbiological and biological performances.

At the structural level, advanced manufacturing techniques should be monitored with new, innovative *in situ* and *ex situ* characterization techniques (e.g., FIB-SEM, X-ray microtomography, NMR cryoporometry, NMR diffusometry, in situ Raman spectroscopy, nanoholotomography) and modelling tools (molecular dynamics simulations, 3D-reversible cellular automata, *ab initio* quantum mechanics density functional theory, meso- and macroscale mechanical, mass transfer and heat transfer models) for aerogel research. Characterization and modelling techniques should be specifically adapted for the screening and validation of aerogel formulations, and for the prediction of the materials performance and processing times (gelation, solvent exchange and drying durations), respectively. When appropriate, new technical specifications/standards should be envisaged to define more reliable characterization methods and protocols.

### 3.3. To Define Manufacturing Protocols for the Safe, Reproducible and Feasible Processing of Aerogels

Research efforts, best working practices, expertise and facilities must be combined together so that design guidelines for aerogels manufacturing on an industrial scale are developed for biomedical and environmental applications. As a result, a “white paper” about health risk and management of aerogel-based materials can be released. Promising aerogel solutions will be thus implemented using rigorous processing designs with full integration of utilities to minimize energy consumptions and to reduce and (when applicable) reuse raw materials for the sake of process economics and of environmental and health-and-safety issues.

The traditional development of aerogels has typically been carried out by material scientists and process engineers with expertise in the technological requirements (management and use of chemicals, operation at high pressures), but not necessarily familiar with the knowledge and the regulation of the production of aerogels for environmental (EPA guidelines) and, particularly, biomedical applications (cGMP and ICH guidelines, FDA and EMA regulations or sterility SAL levels requirements). As an example, no specific sessions on aerogels have been organized within conferences on biomedicine, regenerative medicine or environmental applications so far. For the industrial implementation of aerogels, a set of recommendations on handling and exposure of aerogels should be set for future market and post-market actions on aerogel-based materials used in biomedical and environmental approaches. Health, environmental and exposure risks related to aerogel production should also be evaluated so that compendia of health and safety procedures for production, handling and transport of aerogels can be set. Thus, a selection of adequate personal protective equipment and indoor air cleaning devices for use during aerogels production and handling, of particular relevance in the case of aerogels in the powdered form, can be proposed.

Concerning risk and regulatory issues, there is a paucity of information regarding the health risk assessment and other regulatory aspects on aerogels. A safety regulatory compendium is crucial to advice on the commercialization possibilities of the aerogel-based formulations. Particularly, the framework of health risk assessment of aerogel-based materials for its use in specific biomedical and environmental applications should include health/biota risk and life cycle assessment of aerogels, as well as safety regulatory documentation identifying at least one health risk category. Moreover, a specific panel of tests (e.g., bacterial/viral load, allergen potential, exposure assessment) should be performed for each aerogel-based material and each foreseen application.

## 4. Conclusions

Aerogels are nanostructured highly porous materials with an attractive portfolio of properties (light-weight, high specific surface area and tunable surface chemistry) for emerging applications. Design and engineering of next-generation aerogels with custom tailorable properties is likely to overcome the many of the physical limitations of current biomedical technology and to accelerate both energy efficient materials as well as sustainable development. A number of current biomedical (pharmaceutical technology, regenerative medicine, wound healing) and environmental (sound and thermal insulation, air cleaning, water pollution treatment) demands where aerogel-based products can be advantageous have been identified. However, the main challenge in the development of aerogel-based materials for these fields is the fragmentation between the fundamental research and application needs coupled to an academia-industry-national agencies interlinking to be improved. As a result, there are no mature aerogel-based products in the market for biomedical and environment applications so far, in spite of their high potential. A novel paradigm with a suitable alignment between technological developments of aerogels and the design needs for the intended application through a jointly, multidisciplinary and multisectorial approach is crucial to reach research excellence and to boost the technological progresses on the topic. The awareness of this gap is recently prompting the emergence of scientific-technological networking initiatives assembling international experts from different disciplines (chemical process engineering, biological sciences, materials science, environmental chemistry, drug delivery, regenerative medicine, pharmaceutical technology, physics, pharmacology, toxicology, national agencies) to look for more effective technological and commercial approaches in the development of aerogels [31]. The impact success of these initiatives is based on expanding the audiences by involving the most relevant stakeholders including academia, public research institutions, industry, clinical practitioners, patient organizations, regulatory agencies, NGOs, environmental organizations and the general society.
